# Artificial oxygen carriers rescue placental hypoxia and improve fetal development in the rat pre-eclampsia model

**DOI:** 10.1038/srep15271

**Published:** 2015-10-16

**Authors:** Heng Li, Hidenobu Ohta, Yu Tahara, Sakiko Nakamura, Kazuaki Taguchi, Machiko Nakagawa, Yoshihisa Oishi, Yu-ichi Goto, Keiji Wada, Makiko Kaga, Masumi Inagaki, Masaki Otagiri, Hideo Yokota, Shigenobu Shibata, Hiromi Sakai, Kunihiro Okamura, Nobuo Yaegashi

**Affiliations:** 1 Department of Developmental Disorders, National Institute of Mental Health, National Center of Neurology and Psychiatry, Kodira, Tokyo, Japan; 2 Center for Advanced Biomedical Sciences, Waseda University, Shinjuku-ku, Tokyo, Japan; 3 Image Processing Research Team, RIKEN Center for Advanced Photonics, RIKEN, Wako, Saitama, Japan; 4 Faculty of Pharmaceutical Sciences, Sojo University, Kumamoto, Japan; 5 Department of Pediatrics, St. Luke’s International Hospital, Chuo-ku, Tokyo, Japan; 6 Department of Pediatrics, Japanese Red Cross Medical Center, Tokyo, Japan; 7 Department of Mental Retardation and Birth Defect Research, National Institute of Neuroscience, National Center of Neurology and Psychiatry, Kodaira, Tokyo, Japan; 8 Department of Degenerative Neurological Diseases, National Institute of Neuroscience, National Center of Neurology and Psychiatry, Kodaira, Tokyo, Japan; 9 Department of Chemistry, Faculty of Medicine, School of Medicine, Nara Medical University, Kashihara, Japan; 10 Department of Obstetrics and Gynecology, Tohoku University Hospital, Aoba-ku, Sendai, Japan

## Abstract

Pre-eclampsia affects approximately 5% of all pregnant women and remains a major cause of maternal and fetal morbidity and mortality. The hypertension associated with pre-eclampsia develops during pregnancy and remits after delivery, suggesting that the placenta is the most likely origin of this disease. The pathophysiology involves insufficient trophoblast invasion, resulting in incomplete narrow placental spiral artery remodeling. Placental insufficiency, which limits the maternal-fetal exchange of gas and nutrients, leads to fetal intrauterine growth restriction. In this study, in our attempt to develop a new therapy for pre-eclampsia, we directly rescued placental and fetal hypoxia with nano-scale size artificial oxygen carriers (hemoglobin vesicles). The present study is the first to demonstrate that artificial oxygen carriers successfully treat placental hypoxia, decrease maternal plasma levels of anti-angiogenic proteins and ameliorate fetal growth restriction in the pre-eclampsia rat model.

The molecular mechanism of pre-eclampsia is being increasingly clarified^1^,^2^,^3^ and a hypothesis of pre-eclampsia causing endothelial dysfunction of maternal blood vessels has also been proposed: excess placental secretion of sFlt-1 (soluble fms-like tyrosine kinase, also known as soluble vascular endothelial growth factor (VEGF) receptor 1) and sEng (soluble Endoglin) (two endogenous circulating anti-angiogenic proteins) inhibits VEGF and transforming growth factor (TGF)-β1 signaling respectively in the vasculature, resulting in endothelial cell dysfunction, including decreased prostacyclin, nitric oxide (NO) production and release of procoagulant proteins. The release of sFlt1 and sEng and other inflammatory mediators seems to be induced by maternal factors such as angiotensin II type I receptor activating autoantibodies (AT1-AA), immunologic factors, and oxidative stress^3^. These molecular mechanisms are considered to induce insufficient trophoblast invasion into the decidua portion of the placenta, resulting in incomplete, narrow placental spiral artery remodeling, leading to hypoxic conditions in the placenta. The exact mechanisms responsible for the pathogenesis of pre-eclampsia, however, still remain unclear and further research on its pathogenic mechanisms and clinical treatments is expected.

Possible applications of artificial oxygen carriers (hemoglobin vesicles) for treatment of hypoxia-induced pathology such as brain ischemia have been recently suggested by animal models. The advantages of hemoglobin vesicles over usual red blood cells are that it can provide an effective supply of oxygen even to the pathogenic narrow capillary vessels because of the carriers’ nano-scale size and its absence of blood-type antigens^4^,^5^. The same strategy can be also applied to the placental spiral arteries in pre-eclampsia, in which artificial oxygen carriers treat fetal hypoxia by effectively supplying oxygen, passing through pathogenic narrow spiral arteries in the placenta induced or already established by pre-eclampsia.

To test this hypothesis, we used a rat pre-eclampsia model, in which continuous administration of the NO synthetase inhibitor, N^G^-nitro-L-arginine methyl ester (L-NAME) has been confirmed to induce narrow spiral artery formation, leading to maternal hypertension, placental apoptosis, increased serum tumor necrosis factor (TNF)-α, and fetal hypoxia - all of which have been implicated as being pathophysiological features of pre-eclampsia^6^,^7^. In this model, we hypothesize that continuous intravenous administration of hemoglobin vesicle (HbV), when added to an L-NAME-induced state of chronic NO inhibition during pregnancy, will treat placental and fetal hypoxia and improve fetal development. In this study, we applied to the pre-eclampsia model rat a daily HbV dose of 200 mg/kg/day, which has been proven to be the most effective and safe dosage to rescue hypoxic tissues in animal models in previous studies^4^,^5^,^8^.

## Results

### Change in maternal blood pressure during gestational period

**Figure 1:**
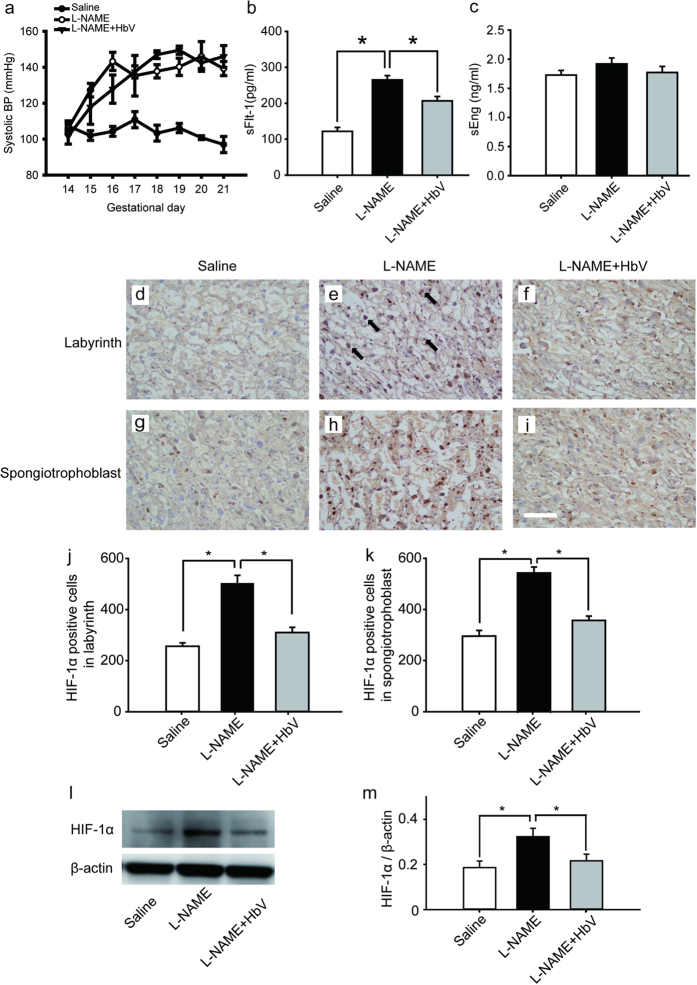
Effects of hemoglobin vesicle (HbV) infusion on maternal blood pressure, sFlt-1/sEng production and placental hypoxia in pregnant rats. (**a**) Chronological changes in systolic blood pressure during pregnancy in saline (control), L-NAME-only treated and L-NAME + HbV treated pregnant rats. Closed circles, saline control pregnant rats; open circles, L-NAME-only treated pregnant rats; closed triangles, L-NAME + HbV treated pregnant rats. The data for each group are expressed as mean values ± s.e. (n = 5). Significant difference between control and L-NAME-only treated rats or L-NAME + HbV treated rats was observed (*P < 0.05, two-way ANOVA, Dunette). (**b**)The plasma sFlt-1 levels in L-NAME-only treated rats were significantly higher compared with those in saline control pregnant rats or L-NAME + HbV treated rats. **(c**)There was no statistical significance in the plasma sEng among the three groups. Data are expressed as mean ± s.e. (*P < 0.05, one-way ANOVA, Dunette). HIF-1α (brown signal) shows stronger signal activity in the labyrinth and spongitrophoblast of the L-NAME-only treated group (**e**), (**h**) compared with the saline control group (**d**), (**g**) or the L-NAME + HbV treated group (**f**), (**i**). Arrows in (**e**) indicate representative HIF-1α positive cells (dark brown cells). Scale bar: 300 μm. Quantification of the HIF-1α-positive cells in the labyrinth (**j**) and spongitrophoblast (**k**). **(l**) Western blot analysis of HIF-1α in the placental tissues from saline, L-NAME, and L-NAME + HbV treated pregnant rats. (**m**) Data quantification of panel (**l**). Western blot was quantified and expressed as the densitometric ratio of HIF-1α/β-actin (n = 5 for each group).

In the rat pre-eclampsia model, chronological changes in systolic blood pressure (SBP) levels were measured (Fig. 1a). 50 mg/day of L-NAME was intravenously infused through a catheter in the jugular vein for 7 consecutive days between gestational day 14 (G14) and G21. The mean SBP at G14 (prior to the start of this experiment) was 107.3 ± 2.9 mmHg (mean ± s.e.) in the control pregnant rats and 106 ± 6.8 mmHg in the group of pregnant rats awaiting L-NAME treatment. Once the L-NAME was administered, the mean SBP of the pregnant rats in this treated group gradually increased by day 16 of pregnancy. This elevation in SBP was maintained until day 21 of pregnancy. A similar increase in mean SBP level was also observed in the group of pregnant rats which received both 50 mg/day of L-NAME and 200 mg/kg/day of hemoglobin vesicles (the L-NAME + HbV treated group). No statistical difference in SBP between the L-NAME-only treated and L-NAME + HbV treated groups was observed between G18 and G21.

### Hemoglobin vesicles decreased sFlt-1 plasma levels in maternal blood

We also examined the effect of hemoglobin vesicles (HbV) on production of sFlt-1 and sEng, candidate molecules for the cause of pre-eclampsia, in the placenta, by comparing the plasma levels of sFlt-1 and sEng among the three groups: saline (control), L-NAME, and L-NAME + HbV treated groups, by ELISA. Pregnant control animals had a significantly lower level of sFlt-1 (122 pg/ml ± 11) than L-NAME-only (265 pg/ml ± 12) and L-NAME + HbV(207 pg/ml ± 12) treated animals during pregnancy (Fig. 1b, P < 0.05. one-way ANOVA, Dunette). In addition, L-NAME-only treated animals had a higher level of sFlt-1 than L-NAME + HbV treated animals. However, there was no statistically significant difference in the plasma sEng between the three groups (Fig. 1c, one-way ANOVA, Dunette). This data suggest that HbV contributed to a decrease in sFlt-1 production by reducing hypoxic conditions in the placenta. Maternal BP, however, continued to remain high as L-NAME, a nitric oxide synthase (NOS) inhibitor which directly regulates nitric-oxide (NO) mediated vasoconstriction, is downstream of sFlt-1 and sEng.

### Hemoglobin vesicles improved placental hypoxic conditions

Next, to confirm the placental hypoxic conditions in the rat pre-eclampsia model, we examined hypoxic-inducible factor 1α (HIF-1α) expression in the placenta. Figure 1d–i shows HIF-1α in the labyrinth (d–f) and spongiotrophoblast (g–i) respectively in fetuses whose mother rats received continuous infusions of saline (Fig. 1d,g), L-NAME (Fig. 1e,h) or L-NAME + HbV (Fig. 1f,i) from G14 through G21. In the labyrinth, HIF-1α positive cells were significantly increased in L-NAME-only treated group (509.8 cells ± 31.1) than saline (control) (255.8 cells ± 13.4) or L-NAME + HbV treated (309.9 cells ± 20.6) groups (Fig. 1j, P < 0.05. one-way ANOVA, Dunette). In the spongiotrophoblast also, HIF-1α positive cells were significantly increased in L-NAME-only treated group (537.3 cells ± 22.8) than saline (289.3 cells ± 21.4) or L-NAME + HbV treated (349.2 cells ± 16.8) groups (Fig. 1k, P < 0.05. one-way ANOVA, Dunette). Western blot also showed that the HIF-1α expression in the whole placenta was significantly higher in the L-NAME-only treated group than the saline or L-NAME + HbV treated groups (Fig. 1l,m). The data indicate that HbV treatment on pregnant mother rats rescued hypoxic conditions in the placenta.

### Evaluation of fetal hypoxic conditions by bioluminescence

**Figure 2:**
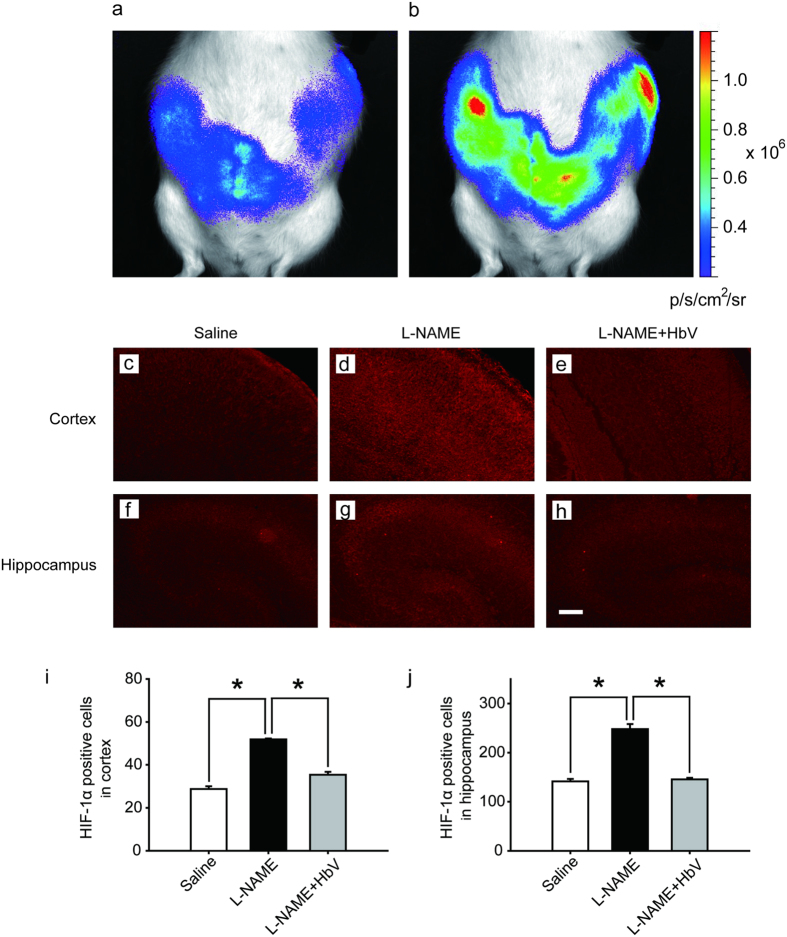
Effects of hemoglobin vesicle (HbV) infusion on fetal hypoxia. Photon flux (p/s/cm2/sr) from the heterozygous Rosa26::luc fetuses is displayed according to the scale bar at the right side. Compared to the basal state of saline injection (**a**), 60-second exposures show that light emission increased after an acute HbV injection (**b**). The cortex and hippocampus of G21 fetuses from the pregnant rats treated with saline ((**c**, **f**) n = 5), L-NAME ((**d**,**g**) n = 5), or L-NAME + HbV ((**e**,**h**) n = 5) were shown respectively. Maternal L-NAME injection induced fetal brain hypoxia, as indicated by more extensive HIF-1α positive staining at the cortex (**d**) and hippocampus **(g**) in fetal brain compared to the same areas of fetal brains from saline (**c**) (**f**) or L-NAME + HbV (**e**) (**h**) treated mothers. Note that HbV attenuated L-NAME-induced fetal brain hypoxia. Quantification of the HIF-1α-positive cells in the cortex (**i**) and hippocampus (**j**). Data are expressed as mean ± s.e. (*P < 0.05, one-way ANOVA, Dunette). Scale bar: 100 μm.

Finally, we investigated whether HbV can rescue fetal hypoxia and improve fetal development. To visually evaluate fetal hypoxia, the hypoxic levels of Rosa26::Luc rat fetuses in the pregnant uterus were examined by the bioluminescence *in vivo* (Fig. 2a,b)^9^. Rosa26 gene is known to express itself ubiquitously in all tissues. Thus, the luciferase gene, which is fused to the endogenous Rosa26 gene promoter, can be monitored throughout the whole body of Rosa26::Luc rats by *in vivo* imaging. Rosa26::Luc rat fetuses were produced by mating a wild-type female Wistar rat with a male homozygous Rosa26::Luc rat, resulting in bioluminescence being detectable only from heterozygous Rosa26::Luc rat fetuses and not from the mother rat. Since firefly luciferase requires oxygen, adenosine triphosphate (ATP), and luciferin to emit light, the bioluminescence of Rosa26::Luc rat fetuses was expected to be elevated by increased oxygen supply to the placentas by HbV. The bioluminescence of the fetuses was assessed after continuous syringe pump infusion of luciferin (0.01 M luciferin, 0.5 ml/h) into the pregnant rats through the catheter in the juglar vein. The bioluminescence levels of the fetuses of L-NAME-only treated mother rats became 1.71 times brighter after acute HbV injections of 0.6ml, which is equivalent to the volume of a daily HbV injection, than after acute saline injections of 0.6 ml (n = 5, P < 0.05. one-way ANOVA). Figure 2b demonstrates a representative data of the increased bioluminescence levels of the fetuses after an HbV injection, indicating that L-NAME-induced fetal hypoxia was rescued by an acute HbV injection.

### Evaluation of hypoxic conditions of the fetal brain

The hypoxic conditions in the fetus were evaluated by HIF-1α expression in the fetal brain. Figure 2c–h show HIF-1α in the cortex (c–e) and hippocampus (f–h) respectively in fetuses whose mother rats received continuous infusions of saline (Fig. 2c,f), L-NAME (Fig. 2d,g) or L-NAME + HbV (Fig. 2e,h) from G14 through G21. In the cortex, HIF-1α positive cells were significantly increased in L-NAME-only treated group (51.9 cells ± 0.4) than saline (28.7 cells ± 1.3) or L-NAME + HbV treated (35.4 cells ± 1.3) group (Fig. 2i, P < 0.05. one-way ANOVA, Dunette). In the hippocampus also, HIF-1α positive cells were significantly increased in L-NAME-only treated group (248.0 cells ± 10.3) than saline (141.1 cells ± 5.0) or L-NAME + HbV treated (145.5 cells ± 3.3) groups (Fig. 2j, P < 0.05. one-way ANOVA, Dunette). The data indicate that HbV treatment to pregnant mother rats rescued hypoxic conditions in the fetal brain.

### Hemoglobin vesicles protected fetal brain apoptotic damage from hypoxia

**Figure 3:**
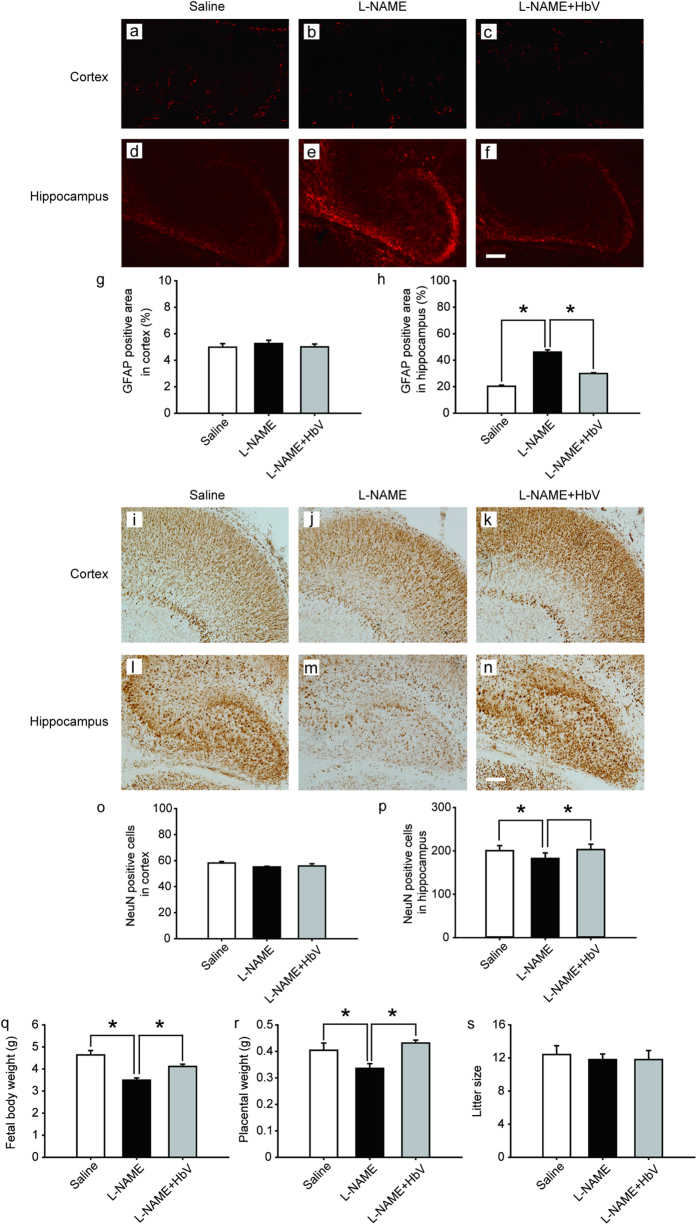
Effects of hemoglobin vesicle (HbV) infusion on fetal brain and body growth under hypoxic conditions. The cortex and hippocampus of G21 fetuses from the pregnant rats treated with saline ((**a**,**d**) n = 5), L-NAME (**b** and **e**, n = 5), or L-NAME + HbV (**c** and **f**, n = 5) were shown respectively. The GFAP positive staining in the hippocampus of fetuses (**e**) from L-NAME treated mothers was stronger compared with those from the saline treated mothers (**d**) or L-NAME + HbV treated mothers (**f**). This indicated that maternal L-NAME injection induced fetal brain astrogliosis and HbV reduced the L-NAME-induced fetal brain damage. Quantification of the GFAP-positive area in the cortex (**g**) and hippocampus (**h**). Data are expressed as mean ± s.e. (*P < 0.05, one-way ANOVA, Dunette). The number of NeuN positive cells in the hippocampus of fetuses (**m**) from L-NAME treated mothers was smaller compared with that in the saline treated mothers (l) or L-NAME + HbV treated mothers (**m**). This indicated that maternal L-NAME injection induced neural damage in the fetal brain while HbV reduced the L-NAME-induced fetal brain damage. Quantification of the NeuN-positive cells in the cortex (**o**) and hippocampus (**p**). Data are expressed as mean ± s.e. (*P < 0.05, one-way ANOVA, Dunette). The weight gains of rat fetuses (**i**) and the placentas (**j**) after chronic HbV or saline infusion at a dose rate of 2 ml/kg/day (n = 5 for each group; value: average ± s.e.) for 7 days. No significant differences in litter size (**k**) were observed among the three groups. Note that HbV rescued the fetal and placental weights in L-NAME + HbV treated groups compared those in L-NAME-only treated groups. Scale bar: 100 μm.

To evaluate fetal brain apoptotic damage from hypoxia, we examined reactive astrogliosis using glial fibrillary acidic protein (GFAP) and NeuN immunostaining^10^,^11^,^12^. GFAP-positive areas in the cortex showed no statistical difference among the three grous: saline, L-NAME-only and L-NAME + HbV treated groups (one-way ANOVA, Dunette, Fig. 3g). In contrast, GFAP-positive areas in the dentate regions of the hippocampus showed that L-NAME-only treated group (46.1% ± 1.7, Fig. 3e) induced a significant increase in GFAP expression compared to saline (20.3% ± 0.9, Fig. 3d) and L-NAME + HbV (30.0% ± 0.6, Fig. 3f) treated groups (one-way ANOVA, Dunette, P < 0.05, Fig. 3h), indicating that HbV treatment rescued fetal hippocampus apoptotic damage from hypoxia. Likewise, NeuN-positive cells in the cortex showed no statistical difference among the three groups (one-way ANOVA, Dunette, Fig. 3o). In contrast, NeuN -positive cells in the dentate regions of the hippocampus showed that L-NAME-only treated group (180.3 cells ± 12.7, Fig. 3m) induced a significant decrease in NeuN -positive cells compared to saline (198.2 cells ± 11.7, Fig. 3l) and L-NAME + HbV (200.6 cells ± 12.3, Fig. 3n) treated groups (one-way ANOVA, Dunette, P < 0.05, Fig. 3p), also indicating that HbV treatment rescued fetal hippocampus apoptotic damage from hypoxia.

### Hemoglobin vesicles improved fetal growth

HbV treatment on L-NAME treated pregnant mother rats also ameliorated fetal and placental growth restriction without changing litter sizes (Fig. 3i,j,k). HbV infusion reversed L-NAME-induced reduction of average fetal body weight of the L-NAME-only treated group (3.5 g ± 0.1) to 4.1 g ± 0.1 in L-NAME + HbV treated group (Fig. 3i).

## Discussion

The present study reports two novel findings regarding the effects of artificial oxygen carriers (hemoglobin vesicles: HbV) in the rat model of pre-eclampsia. Firstly, we are the first to report that HbV ameliorates the fetal growth restriction and brain apoptotic damage in the pre-eclampsia rat model. In contrast to previous therapy strategies using chronic infusion of adrenomedullin and VEGF-121, failed to rescue decrease in fetal body weight gain^13^,^14^. The different results between the present and previous studies may relate to the extent that each therapy rescues hypoxic conditions in the placenta, which also leads to reduction of fetal hypoxia. As shown in Figs 1 and 2, HIF-1α expressions in both the placenta and fetal brain tissues were decreased by HbV infusion, indicating that HbV rescued fetal hypoxia by increasing oxygen supply through the placenta. This is also supported by *in vivo* imaging of Rosa26::luc fetuses, in which the bioluminescence of fetuses is increased by acute HbV injections, indicating that fetal hypoxic conditions are rescued by oxygen supply provided by HbV (Fig. 2a,b). In addition, our data indicate that brain injury, reactive astrogliosis, in fetal brain tissue due to hypoxia was prevented by chronic HbV infusion (Fig. 3a–p).

Second, we also found that the serum level of sFlt-1 was decreased in the L-NAME + HbV treated group compared to the L-NAME-only treated group. This is consistent with previous reports stating that placental ischemia/ hypoxia results in elevated circulating levels of sFlt-1^2^ since chronic HbV infusion seems to have directly rescued placental hypoxia in the present study. Our data also matched the recent data in the same rat pre-eclampsia model, in which chronic L-NAME infusions increased sFlt-1 but induced no change in sEng in pregnant rats^15^. The data indicates a possible new treatment using HbV for pre-eclampsia, in which excess placental secretion of sFlt-1 and sEng inhibits VEGF and TGF-β1 signaling respectively in the vasculature, resulting in decreased endothelial NO production. Future studies, such as applying HbV to another pre-eclampisa animal model induced by sFlt-1 and sEng agonists, or systemic measurement of all pre-eclampisa-related substances after HbV treatment, would be required to reconfirm the effects of HbV on the pre-eclampsia mechanism.

In humans, minimum capillary diameters have been reported to be around 8 μm, approximately equal in size to human red blood cells^16^. Previous studies have indicated that, even among different species, all mammals have similar capillary diameters since their peripheral tissues have the same physiological properties necessary for exchanging gas, nutrients and metabolic wastes between capillary vessels^17^. These data support the possibility that application of 250 nm-diameter hemoglobin vesicles will effectively treat pre-eclampsia in humans by supplying oxygen through narrow placental vessels induced by pre-eclampsia. Although further investigations are required to elucidate whether the same effects would occur in humans, our results are the first to report the treatment effect of HbV on fetuses and placentas in a pre-eclampsia animal model, and have also provided important information for possible HbV clinical applications for addressing fetal hypoxic conditions induced by a pathogenic placenta during pregnancy. In the future, chronic infusion of HbV could be a potentially effective noninvasive therapy for delaying or even alleviating the need for Ceasarean sections, the last-resort therapy for pre-eclampsia.

## Methods

### Animals and experimental procedures

Animal care and use were reviewed and approved by the Committee for the National Center of Neurology and Psychiatry (approval#2011023) and all procedures were carried out in accordance with the approved guidelines. 8-week-old female Wistar rats were purchased from Clea Japan (Tokyo, Japan) and housed with food and water ad libitum in a temperature controlled room (23 °C) on a 12:12-h light/dark cycle. After one week of habituation, the 9-week-old female rats were mated overnight during the pro-estrus period and timed pregnant rats (vaginal smear positive, gestational day 0 (G0); term, G22) were used for the following experiments. At G10, rats were anesthetized by isoflurane inhalation and then pentobarbital sodium i.p. and a catheter was surgically secured in the internal jugular vein in all the rats. During 7 consecutive days between G14 and G21, the rats received either saline (n = 5), 50 mg/day of L-NAME (Sigma, St.Louis, MO) (n = 5), or 50 mg/day of L-NAME + 200 mg/kg/day of hemoglobin vesicle (HbV) (n = 5) via the catheter. For the L-NAME + HbV group, HbV was infused at a dose rate of 2 ml/kg body weight with an injection rate of 1 ml/min. The total volume of HbV infused into each rat over 7 days reached 14 ml/kg, which was equal to 25% of the actual blood volume of the rat (56 ml/kg). The total infused HbV is calculated to be 1400 mg Hb/ kg. The maternal blood pressure was also measured from G14 to G21 once a day. At G21, one day before term, the pregnant rats were sacrificed and the fetuses, placenta, and maternal blood were sampled for assessment of placental and fetal hypoxia, placental and fetal weights, and plasma sFlt-1 and sEng, markers of pre-eclampsia.

### Preparation of Hemoglobin Vesicle (HbV) Suspension

The test fluid, the HbV suspension, was prepared under sterile conditions as reported previously^18^,^19^. Human Hb was purified from outdated donated blood provided by the Japanese Red Cross Society (Tokyo, Japan) through pasteurization and nanofiltration. The encapsulated Hb contained pyridoxal 5-phosphate (Aldrich Chemical Co., Milwaukee, WI) as an allosteric effector. The lipid bilayer was composed of a mixture of 1,2-dipalmitoyl-sn-glycero-3-phosphatidylcholine, cholesterol, 1,5-O-dihexadecyl-N-succynyl-L-glutamate (Nippon Fine Chemicals Co., Osaka, Japan), and 1,2-distearoyl-sn-glycero-3-phosphatidylethanolamine-N-PEG5000 (NOF Co., Tokyo, Japan) at a molar ratio of 5:4:1:0.03. HbVs were suspended in a physiological salt solution and deoxygenated with bubbling N_2_ for storage^20^. The physicochemical parameters of the HbV are as follows: particle diameter, 252 ± 53 nm; [Hb], 10 g/dl; [lipids], 6–7 g/dl; and oxygen affinity P_50_, 25–28 Torr.

### Measurement of Maternal Blood Pressure (BP)

From G14 to G20, BP values of the rats were recorded on a Model MK-2000ST recorder (Muromachi Kikai Co., Ltd., Tokyo, Japan) once every day. The pregnant rats were placed in plastic restrainers. A cuff with a pneumatic pulse sensor was attached to the tail. Each recorded BP value was averaged from at least three consecutive readings obtained from each rat within one day^21^.

### Plasma sFlt-1 and sEng assay

Blood was collected for subsequent assays into Vacutainer EDTA container tubes (Terumo). Circulating sFlt-1 and sEng concentrations were measured using commercial ELISA kits available from R&D Systems (Quantikine) and USCN Life Science Inc. respectively, following the manufacturer’s directions^15^,^22^.

### Immunohistochemical analysis of the fetal brain and the placentas

At G21, the animals were sacrificed and the fetuses and placentas were resected for weight measurements and immunohistochemical staining. The placentas were fixed in 10% formaldehyde neutral buffer solution immediately after removal, and embedded in paraffin. The sections were cut at a thickness of 5 μm. The fetal brains were fixed in 4% PFA (Wako Pure Chemicals) overnight and immersed in 30% sucrose, then stored at −80 °C. The frozen sections of the fetal brains were cut at a thickness of 25 μm.

Immunohistochemical analyses were performed to detect HIF-1α in the placentas and HIF-1α, GFAP and NeuN in the fetal brains. Placenta sections selected for immunostaining were incubated at room-temperature for 30 minutes with HIF-1α antibody (1:200, Novus, Cambridge, UK) and followed by a 30-min room-temperature incubation in anti-rabbit secondary antibody at 1:100 (Vector Laboratories, Burlingame, CA) and streptavidin-peroxidase complex at 1:50 (Vectastain ABC Kit; Vector Laboratories).

Brain sections selected for fluorescent immunostaining were incubated overnight at 4 °C with HIF-1α antibody (1:200, Novus, Cambridge, UK) and GFAP antibody (1:300, DAKO, Produktionsvej, Denmark). Then the sections were incubated in Goat anti-rabbit secondary antibody Alexia 594 (1:300) for 60 minutes at room-temperature. Finally, the sections were rinsed with PBS and cover slipped with mounting medium (H-1000, Vectashield® Mounting Medium; Vector Laboratories, Burlingame, CA) to reduce fading of the immunofluorescence. Brain sections selected for NeuN immunostaining were incubated at room-temperature for 30 minutes with NeuN antibody (1:100, Millipore, Billerica, USA) and followed by a 30-min room-temperature incubation in anti-mouse secondary antibody at 1:100 (Vector Laboratories, Burlingame, CA) and streptavidin-peroxidase complex at 1:50 (Vectastain ABC Kit; Vector Laboratories). The immunostained sections were observed under an IX71 inverted microscope (Olympus, Tokyo, Japan).

The number of HIF-1α and NeuN positive cells or GFAP positive areas in the placenta or fetal brain were quantified with the aid of Image J 1.45 s (National Institutes of Health, USA). In the placenta, the counts in each region of interest were obtained from the average counts from all coronal sections of 3 mm thick tissue from the center to the periphery of the placenta, which includes both the labyrinth and spongiotrophoblast. The counting boxes for both the labyrinth and spongiotrophoblas were 1.6 mm × 1.0 mm. In fetal brain sections, the counts in each region of interest were obtained from the average of all coronal sections between the bregma levels +1.0 mm and −2.0 mm, which covered the major portions of the anterior cingulated cortex and hippocampus. The counting box for the cortex, a square of 130 μm × 130 μm was placed at the anatomical structures of interest. For hippocampus, the box was placed corresponding to dentate gyrus with a square of 390 μm × 330 μm.

### Western blot analysis of the placentas

For Western blot analysis, whole placentas were homogenized in lysis buffer [50 mmol/l Tris, 150 mmol/l NaCl, 2 mmol/l EDTA, 1% Triton-x100, 0.1% SDS and 1 tablet/10 ml protease inhibitor (complete mini, EDTA-free, Roche) in distilled water]. Samples were centrifuged (12000 rpm) at 4 °C for 15 min and the supernatant was separated and stored at −80 °C. The protein content was analyzed by commercial protein assay (BCA protein assay, Pierce Biological, Rockford, IL). Protein (10 μg) was run on a 7.5% SDS-polyacrylamide gel, transferred to a PVDF transfer membrane, blocked in commercial blocking buffer (Blocking One, nacalai tesque, Kyoto, Japan) for 90 min at room temperature, and incubated overnight at 4 °C with the polyclonal rabbit anti-HIF-1α antibody (1:1000, Novus, Cambridge, UK). After washing, blots were incubated with a horseradish peroxidase-conjugated secondary antibody (1:2500, GE, Buckinghamshire, UK) for 1 h at room temperature, followed by ECL-detection. β-actin was used as a loading control. Relative protein levels were quantified by scanning densitometry and analyzed by Image J 1.45s (National Institutes of Health, USA).

### *In Vivo* imaging for evaluating fetal hypoxia

To visually evaluate fetal hypoxia, the hypoxic levels of Rosa26::Luc rat fetuses in the pregnant uterus were examined by bioluminescence *in vivo*. Wild-type female rats were mated to homozygous Rosa26::Luc transgenic males to obtain heterozygous Rosa26::Luc transgenic fetuses. Rosa26 promoter has initially been identified by random retroviral gene trapping in mouse embryonic stem cells and can drive expression of reporter genes in all cells throughout embryonic development and in adult tissues. Since firefly luciferase requires oxygen, ATP, and luciferin to emit light, the bioluminescence of Rosa26::Luc rat fetuses is expected to be elevated by increased oxygen supply to the placentas induced by HbV^9^.

For *in vivo* imaging, four L-NAME-only treated pregnant rats were anesthetized by 150 μL pentobarbital sodium i.p. and D-Luciferin (0.01 M luciferin, 0.5 ml/h; Xenogen, Cranbury, NJ) was injected through the catheter in the juglar vein. Then, dams were reanesthetized by 2.5–3% isofluorane vaporized in 1 L/min O_2_, and their abdominal portion was imaged beginning 10 minutes post injection of D-Luciferin using an IVIS Imaging System and a charge-coupled device camera (15 cm field of view, binning 8, 1/f stop, open filter; 100 series; Xenogen). In utero bioluminescence from the abdomens of the dams was analyzed using Living Image 2.20 (Xenogen) and Igor (Wavemetrics, Portland, OR) software. Total photon flux (photons/s) was measured from the 1 minute exposure at the sampling time point^23^.

### Data Analysis

The results are shown as the mean ± standard error (s.e.). The maternal blood pressures among the groups were compared by a two-way ANOVA test. Comparisons between the groups of plasma sFlt-1 and sEng, placental HIF-1α, and fetal brain expressions of HIF-1α and GFAP were performed using a one-way ANOVA. SPSS 17.0 (SPSS Inc. Chicago, IL, USA) software was used for the analyses. The level of statistical significance was set at P < 0.05.

## Additional Information

**How to cite this article**: Li, H. *et al.* Artificial oxygen carriers rescue placental hypoxia and improve fetal development in the rat pre-eclampsia model. *Sci. Rep.* **5**, 15271; doi: 10.1038/srep15271 (2015).
